# Alterations in Antioxidant Status and Erythrocyte Properties in Children with Autism Spectrum Disorder

**DOI:** 10.3390/antiox12122054

**Published:** 2023-11-28

**Authors:** Tomas Jasenovec, Dominika Radosinska, Katarina Jansakova, Maria Kopcikova, Aleksandra Tomova, Denisa Snurikova, Norbert Vrbjar, Jana Radosinska

**Affiliations:** 1 Institute of Physiology, Faculty of Medicine, Comenius University in Bratislava, Sasinkova 2, 811 08 Bratislava, Slovakia; tomas.jasenovec@fmed.uniba.sk (T.J.); katarina.jansakova@fmed.uniba.sk (K.J.); maria.kopcikova@fmed.uniba.sk (M.K.); aleksandra.tomova@fmed.uniba.sk (A.T.); 2 Institute of Medical Biology, Genetics and Clinical Genetics, Faculty of Medicine, Comenius University in Bratislava, Sasinkova 4, 811 08 Bratislava, Slovakia; dominika.radosinska@fmed.uniba.sk; 3 Centre of Experimental Medicine, Slovak Academy of Sciences, Institute for Heart Research, Dúbravská Cesta 9, 841 04 Bratislava, Slovakia; usrddena@savba.sk (D.S.); norbert.vrbjar@savba.sk (N.V.)

**Keywords:** autism, oxidative stress, erythrocytes, deformability, nitric oxide, Na,K-ATPase, erythrocyte morphology

## Abstract

Erythrocytes are responsible for the transport of oxygen within the organism, which is particularly important for nerve tissues. Erythrocyte quality has been shown to be deteriorated in oxidative stress conditions. In this study, we measured the same series of oxidative stress markers in plasma and erythrocytes to compare the differences between neurotypical children (controls) and children with autism spectrum disorder (ASD). We also focused on erythrocyte properties including their deformability, osmotic resistance, Na,K-ATPase activity, nitric oxide levels and free radical levels in children with ASD and controls. Greater oxidative damage to proteins and lipids was observed in the erythrocytes than in the plasma of ASD subjects. Additionally, antioxidant enzymes were more active in plasma samples from ASD children than in their erythrocytes. Significantly higher nitric oxide level and Na,K-ATPase enzyme activity were detected in erythrocytes of ASD individuals in comparison with the controls. Changes in oxidative status could at least partially contribute to the deterioration of erythrocyte morphology, as more frequent echinocyte formation was detected in ASD individuals. These alterations are most probably responsible for worsening the erythrocyte deformability observed in children with ASD. We can conclude that abnormalities in antioxidant status and erythrocyte properties could be involved in the pathomechanisms of ASD and eventually contribute to its clinical manifestations.

## 1. Introduction

Oxidative stress is considered a typical feature of most diseases. The imbalance between the production and elimination of free radicals leads to an elevation of reactive molecules that circulate in the blood. Circulating radicals come into contact with red blood cells (RBCs), which are responsible for the transport of oxygen within the organism. Exposure to oxygen, as well as a limited ability to resist oxidative damage (as RBCs are not real cells), can lead to the deterioration of RBC quality in a variety of pathological conditions, as shown by human studies [1]. RBC functionality is particularly important for nerve tissue because of the continuous and high oxygen requirements of nerve cells. To accomplish this oxygen-delivery function, RBCs are supposed to deform repeatedly during their transport through the capillaries, as the smallest ones are narrower than the diameter of RBCs. The deformability of RBCs is thus crucial for functional microcirculation. In addition to oxidative damage, the ability of RBCs to reversibly deform is influenced and conditioned by proper ion homeostasis, which is mostly ensured by the enzyme Na,K-ATPase, as well as by the production of nitric oxide (NO) and the mechanical properties of the RBC membrane [2].

Autism spectrum disorder (ASD) represents a complex of neurodevelopmental disorders with increasing incidence. ASD remains the focus of many research teams searching for biomarkers, indicating the severity and progression of this disorder or helping to clarify its etiopathogenesis, which is still unclear. ASD is characterized by core symptoms that include problems in social communication and interaction, as well as restricted repetitive patterns of behavior, activities or interests [3]. In addition to these behavioral characteristics, children with ASD typically suffer from a wide range of somatic problems at a higher rate than their neurotypical counterparts, e.g., feeding and gastrointestinal disorders [4,5], immunological disturbances [6], sleep disorders [7], neurological diseases and a variety of others [8,9]. Oxidative stress, implicated in the pathophysiology of multiple disorders, has been shown to be a common denominator of neurodevelopmental diseases including ASD [10,11,12]. It could represent a consequence of the insufficient action of cellular antioxidants [13] or the enhanced production of reactive substances, e.g., due to mitochondrial dysfunction [14,15] in ASD conditions. As mentioned previously, oxidative stress generates unfavorable conditions for the optimal functioning of RBCs, leading to the deterioration of their properties [1]. A decrease in the quality of RBCs could have a significant impact on hemorheology [16], and thus could aggravate the clinical symptoms of multiple disorders due to the suboptimal delivery of oxygen to metabolically active tissues [17]. We have previously proposed that abnormalities in RBC deformability may be involved in the pathomechanisms of ASD and contribute to its clinical manifestations [18]. The aim of this study was to determine a series of oxidative stress markers in blood plasma and in RBCs, as well as to focus on RBC properties including the deformability, Na,K-ATPase activity, and NO and free radical production by RBCs in ASD and neurotypical children. The reasoning for such an approach was based on previously observed indices of oxidative stress and deteriorations of RBC quality in ASD, e.g., see [19,20,21]. However, relatively few studies have focused on the simultaneous determination of the oxidative state in blood plasma and in RBCs, together with parameters affecting RBC functions. Furthermore, not all data that are available in databases show consistent results.

## 2. Materials and Methods

### 2.1. Study Design

We enrolled 36 non-verbal children diagnosed with ASD (32 boys and 4 girls) and 17 neurotypical children (12 boys and 5 girls) in this study. The median age of children with ASD was 3.3 years, ranging from a minimum of 2.7 years to a maximum of 7.6 years, while the neurotypical children had ages ranging from 2.4 to 6.3 years, with a median value of 5.4 years, without significant differences between both groups. ASD diagnosis followed the criteria outlined in the DSM-V (American Psychiatric Association 2013). We administered the Autism Diagnostic Interview-Revised (ADI-R) and the Autism Diagnostic Observation Schedule–second edition (ADOS-2) as our diagnostic tools, as described previously [18]. Children with suspected ASD diagnosis were administered Module 1 of ADOS-2, specifically designed for children over 30 months of age who do not consistently use phrase speech. This module consists of 10 structured tasks to assess social interactions and communication deficits. Each type of behavior has its own score that is needed for the diagnostic algorithm. Participants assigned to the control group were selected from a group of children with no known history of ASD. We also administered the M-CHAT screening questionnaire with their parents. There were no significant scores indicative of ASD. All evaluations and ASD diagnoses were conducted at the Academic Research Center for Autism, Institute of Physiology, Faculty of Medicine, Comenius University in Bratislava. This study received approval from the Ethics Committee of the Faculty of Medicine, Comenius University and University Hospital in Bratislava, Slovak Republic. Informed consent was obtained from all caregivers of the children. The study adhered to the ethical principles outlined in the Declaration of Helsinki.

### 2.2. Blood Processing

Venous blood was drawn into EDTA-containing vacutainer tubes right after the diagnostic procedures. The blood was immediately centrifuged at 1150× *g* for 5 min at 4 °C. The plasma was collected, while the buffy coat and the upper 20% of RBCs were removed. The remaining RBCs were subjected to three washes with saline solution. A portion of the RBCs were subjected to osmotic hemolysis in distilled water (*v*:*v*, 1:19). Plasma and hemolyzed RBCs were stored at −80 °C until further analysis.

### 2.3. Parameters of Oxidative Stress and Antioxidant Status

Oxidative stress markers, antioxidant status and carbonyl stress were detected in both plasma and hemolyzed RBCs. All chemicals were purchased from Merck Sigma-Aldrich (Merck KGaA, Darmstadt, Germany), and analyses were performed by using a Synergy H1 Hybrid Multi-mode Reader machine (Agilent, Santa Clara, CA, USA).

Measurement of oxidative stress markers included the detection of thiobarbituric acid reactive substances (TBARS) as a marker of lipid peroxidation and advanced oxidation protein products (AOPP) as a marker or protein oxidation. For the assessment of TBARS, 20 μL of the samples and 1,1,3,3-tetraethoxypropane used for the calibration curve were mixed in a PCR plate with 30 μL of distilled water, 20 μL of 0.67% thiobarbituric acid prepared with dimethyl sulphoxide and 20 μL of glacial acetic acid. The plate was then incubated for 45 min at 95 °C. After incubation, 100 μL of n-butanol was added into every well, briefly mixed and subsequently centrifuged at 2000× *g* and 4 °C for 10 min. The fluorescence was detected in the upper organic phase (70 μL) transferred into a dark microtiter plate at λ_ex_ = 515 nm and λ_em_ = 553 nm. To measure AOPP, 200 μL of chloramine T used as a standard was incubated for 6 min with potassium iodide and samples were added into a transparent microtiter plate. Twenty microliters of glacial acetic acid was added into both samples and standards and mixed for 2 min. The absorbance was then read at λ = 340 nm [22].

As a marker of antioxidant status, the assessment of ferric reducing antioxidant power (FRAP) was applied according to the method developed by Benzie and Strain [23]. The absorbance of 200 μL of freshly prepared and warmed FRAP reagent composed of acetate buffer, iron (III) chloride hexahydrate solution and distilled water was read as a blank at λ = 593 nm. Then, we added a 20 μL mixture of the samples and iron (II) sulphate heptahydrate used as a standard into a transparent microtiter plate and measured the absorbance at λ = 593 nm.

Carbonyl stress was assessed via the measurement of fructosamine, representing a marker of advanced glycation of proteins [24]. A 20 μL mixture of the samples and standards (1-deoxy-morpholino-D-fructose) was incubated with 100 μL of a 0.25 mmol/L nitroblue tetrazolium (NBT) solution composed of sodium carbonate and 1 mmol/L NBT at 37 °C for 15 min. The whole mixture in a plate was then measured at λ = 530 nm.

The measurement of reduced and oxidized glutathione (GSH/GSSG), and the subsequent calculation of their ratio, was used as an indicator of oxidative stress. A 10 μL mixture of the samples and reduced L-glutathione as a standard was added into a dark microtiter plate, mixed with 10 μL of O-phtalaldehyde solution and PBS with 2.5 mmol EDTA-Na_2_ and incubated at room temperature for 15 min. After incubation, fluorescence was measured at λ_ex_ = 350 nm and λ_em_ = 460 nm. The GSSG detection procedure included the mixing of 25 μL samples and oxidized (-)-glutathione used as a standard with 10 μL of N-ethylmaleimide and incubating the mixture at room temperature for 40 min. Thereafter, 10 μL of the sample/standard mixture was transferred into a dark plate and incubated for 15 min with a 10 μL O-phtalaldehyde solution and 180 μL of 0.1 mmol/L NaOH. Fluorescence was measured at λ_ex_ = 350 nm and λ_em_ = 460 nm.

Additionally, the activity of three important enzymes of cellular antioxidant defense, namely superoxide dismutase (SOD), catalase (CAT) and glutathione peroxidase (GPx), was determined in plasma samples and hemolysates. For CAT, the method presented by Aebi [25] was applied. The sample (10 μL) was incubated for 3 min either with hydrogen peroxide solution or distilled water (100 μL). Then, ammonium molybdate (240 μL, 32.4 mM) was added and absorbance was measured at λ = 374 nm. For SOD and GPx activities, commercial kits were used (CS0009 and MAK437, Merck Sigma-Aldrich, Merck KGaA, Darmstadt, Germany), following the manufacturer’s instructions.

### 2.4. Erythrocyte Parameters Assessment

To evaluate the RBC deformability, we employed a filtration technique, consistent with our previous experiments [26]. In short, RBC deformability was determined as the ratio of the count of RBCs that passed through a membrane filter with pores that were 5 μm in diameter (Ultrafree-MC SV Centrifugal Filter; Merck Millipore Ltd., Tullagreen Carrigtwohill, Ireland) to the initial RBC count before centrifugation. The Sysmex hematological analyzer (Sysmex F-820, Sysmex Corp, Tokyo, Japan) was used to count RBCs as well as to determine the mean cell volume (MCV) and RBC distribution width (RDW-SD). The MCV parameter reflects the size of a single RBC, and RDW indicates the differences in the volume of RBCs. The morphology of RBCs was observed using light microscopy. Ten microliters of whole blood was mixed with 90 µL of physiological solution, and blood smears were prepared from the mixture and processed immediately. RBCs were captured using a microscope (Axiolab 5, Zeiss, Jena, Germany) and classified into three categories, namely normal disc cells, echinocytes I (irregularly shaped RBCs), and echinocytes II (RBCs with spikes), as described previously [27]. On average, 750 RBCs per sample were analyzed.

### 2.5. Fluorescent Microscope Techniques

For the purposes of assessing RBC nitric oxide (NO) production, RBC calcium ion content, as well as reactive oxygen species presence, we utilized the following fluorescent probes: 4,5-diaminofluorescein diacetate (DAF-2 DA, 25 µmol/L, ab145283, Abcam, Cambridge, UK), Fluo-3 AM (4 µmol/L, ENZ52004 Enzo Life Sciences, Inc., Farmingdale, NY, USA) and dichlofluorescein (DCF, 50 μmol/L, D6883, Merck, Darmstadt, Germany). Before incubation with the corresponding probes, washed RBCs were diluted in modified physiological solution (*v*:*v*, 1:19; in mmol/L: NaCl 119, KCl 4.7, NaHCO_3_ 25, MgSO_4_·7H_2_O 1.17, KH_2_PO_4_ 1.18, CaCl_2_·2H_2_O 2.5, Na_2_EDTA 0.03, glucose 5.5, and pH 7.4), whereas for the Fluo 3 AM probe, the solution was adjusted to a slightly higher calcium level, as described previously [28] (in mmol/L: NaCl 116.1, KCl 4.6, NaHCO_3_ 24.4, MgSO_4_·7H_2_O 1.14, KH_2_PO_4_ 1.15, CaCl_2_·2H_2_O 5, Na_2_EDTA 0.03, glucose 5.37, and pH 7.4). The suspension was subsequently treated with a particular fluorescent probe, and the samples were incubated in the dark at 37 °C. After 30 min of incubation, the samples were washed and resuspended in the corresponding solutions. The fluorescence signal was captured using Zeiss Microscope Fluorescence Filter Cube Set 38 HE 489038-9901 (λ_ex_ = 470/40 nm, λ_em_ = 525/50 nm) and the fluorescence microscope Axiolab 5 (Zeiss, Jena, Germany). Quantification of the fluorescent signal intensity was performed using the ZEN 3.4 Blue (Carl Zeiss Microscopy GmbH, Jena, Germany) and ImageJ 1.53e software (National Institutes of Health, Bethesda, MD, USA), while it is expressed in arbitrary units per individual RBC.

### 2.6. Na,K-ATPase Enzyme Kinetics Assessment

The isolation of RBC membranes and the determination of Na,K-ATPase kinetic parameters were performed as described previously [27], while chemicals were purchased from Sigma-Aldrich (St. Louis, MO, USA). Washed RBCs were homogenized in 50 mmol/L TRIS, followed by centrifugation at 13,000× *g* for 30 min at 4 °C. The supernatant was then discarded, and the membrane-containing pellets were subjected to repeated homogenization and centrifugation in 30, 20, and 10 mmol/L TRIS solutions to eliminate residual hemoglobin. Na,K-ATPase activities were measured across a range of Na^+^ concentrations (2–100 mmol/L). RBC membrane proteins (50 µg) were preincubated for 20 min at 37 °C, and ATP (final concentration 8 mmol/L) was subsequently added. The chemical reaction was terminated after 20 min with 12% trichloroacetic acid. Inorganic phosphate generated via ATP hydrolysis was spectrophotometrically determined at λ = 700 nm. The obtained data on Na,K-ATPase activity at each NaCl concentration were used to generate kinetic curves and to derive the kinetic parameters of the Na,K-ATPase enzyme: V_max_ (maximum reaction velocity, i.e., the rate of the reaction at which the enzyme shows the highest turnover) and K_Na_ (the concentration of Na^+^ required for half-maximum activation of the enzyme).

### 2.7. Assessment of Red Blood Cell Osmotic Resistance

The RBC osmotic resistance was determined using the established procedure [29]. A set of NaCl solutions ranging from 0% (i.e., distilled water) to 0.9% were prepared. Washed RBCs were exposed to each NaCl solution for 30 min, followed by centrifugation. In supernatants, the level of hemolysis was determined spectrophotometrically at λ = 540 nm. Supernatants from 0.9% NaCl were considered nonhemolytic, while those from distilled water were used as a reference for 100% hemolysis. Subsequently, the concentration of NaCl resulting in 50% hemolysis (IC_50_) was calculated. A decrease in the IC_50_ value corresponds to an increase in the RBC osmotic resistance.

### 2.8. Statistical Analyses

Outliers were identified via the standardized Grubbs test and subsequently excluded from further analyses. The D’Agostino–Pearson test was employed to assess the data normality. Data are presented either as means ± standard deviations or as medians with interquartile ranges when a non-Gaussian distribution was observed. Statistical significance between groups was assessed using the unpaired t-test when data were normally distributed or Mann–Whitney test when not. The occurrence of different morphologies of RBCs was analyzed using the chi-square test. Significance was set at *p* < 0.05. Data analysis was performed using GraphPad Prism 7.02 software (GraphPad Software, San Diego, CA, USA).

## 3. Results

### 3.1. Parameters of Oxidative Stress and Antioxidant Status

Focusing on blood plasma, higher concentrations of fructosamine, a marker of early protein glycation, were observed in children with ASD compared with neurotypical children. The investigation of glycation parameters has been suggested as clinically relevant not only for the management of diabetes mellitus, but also for the panel of metabolic markers for the diagnosis of ASD [30]. In addition, higher activities of antioxidant enzymes-catalase and glutathione peroxidase were observed in children with ASD compared with neurotypical children.

In RBCs, higher levels of markers indicating oxidative damage to proteins (AOPP) and lipid peroxidation (TBARS) were found in children with ASD when compared with the controls.

The decrease in the plasma GSH/GSSG ratio (general marker of oxidative stress indicating the thiol–disulfide balance) and the increase in catalase activity in RBCs did not reach the level of statistical significance (*p* = 0.06 for both). All of the determined parameters with statistics are available in Table 1.

### 3.2. Erythrocyte Parameters

**Figure 1:**
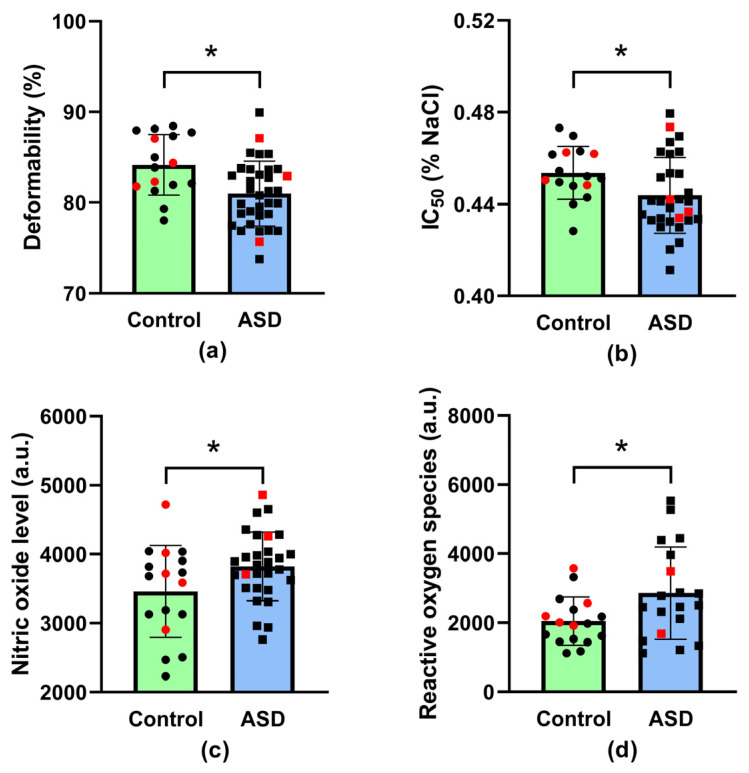
Erythrocyte deformability (**a**), erythrocyte osmotic resistance, presented as NaCl concentration at which 50% of hemolysis occurs (IC_50_) (**b**), nitric oxide level (**c**) and the level of reactive oxygen species (**d**) in erythrocytes of neurotypical (i.e., control) children (*n* = 16–17) and children with autism spectrum disorder (ASD, *n* = 19–34). * *p* < 0.05 versus control group. Data are presented as means ± standard deviations. Statistical significance between groups was assessed using the unpaired *t*-test. Red dots indicate female study participants.

Regarding the alteration in the size of a single RBC expressed by MCV and the variability in the size of RBCs-RDW, there were no differences between neurotypical children and children with ASD (for MCV: 79.9 ± 3.1 fL in neurotypical vs. 79.5 ± 4.4 fL in ASD, *p* = 0.76; for RDW-SD: 37.3 ± 2.4 fL in neurotypical vs. 38.4 ± 3.2 fL in ASD, *p* = 0.74). Both parameters are a part of the standard complete blood count, while their deviations are related to nutritional deficiencies, certain inflammatory diseases as well as problems with the RBC production in general [31]. The lack of changes in the MCV and RDW parameters indicates the absence of significant problems with the production of RBCs, so we can exclude a more obvious nutrient deficiency that interferes with erythropoiesis in our ASD children. However, RBC deformability was lower (*p* = 0.017, Figure 1a) and the osmotic resistance of RBCs was higher (*p* = 0.026, Figure 1b) in children with ASD compared with neurotypical ones. The nitric oxide concentration (*p* = 0.04) as well as the level of reactive oxygen species (*p* = 0.03) were also higher in RBCs from children with ASD compared with neurotypical children (Figure 1c,d). There was no significant difference in RBC Ca^2+^ content between the groups (in arbitrary units: 2491 ± 607 in neurotypical vs. 2309 ± 660 in ASD, *p* = 0.38).

**Figure 2:**
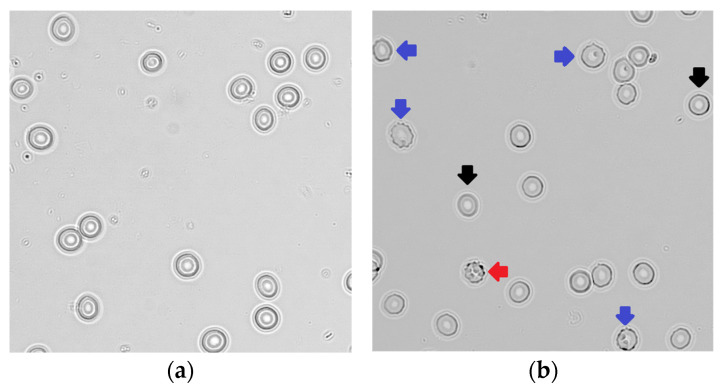
Representative photographs of erythrocytes in neurotypical children (**a**) and children with autism spectrum disorder (**b**). Black arrows: normal erythrocytes; blue arrows: echinocyte I (irregularly contoured erythrocytes); red arrow: echinocyte II (erythrocyte with spicules).

The RBC morphology was significantly altered in children with ASD, as type I and II echinocytes were more frequent compared with neurotypical controls (*p* < 0.0001). The percentages of individual shapes of RBCs in peripheral blood smears are presented in Table 2, while representative photographs of the RBC morphology are shown in Figure 2.

### 3.3. Na,K-ATPase Enzyme Kinetics in Erythrocyte Membranes

**Figure 3:**
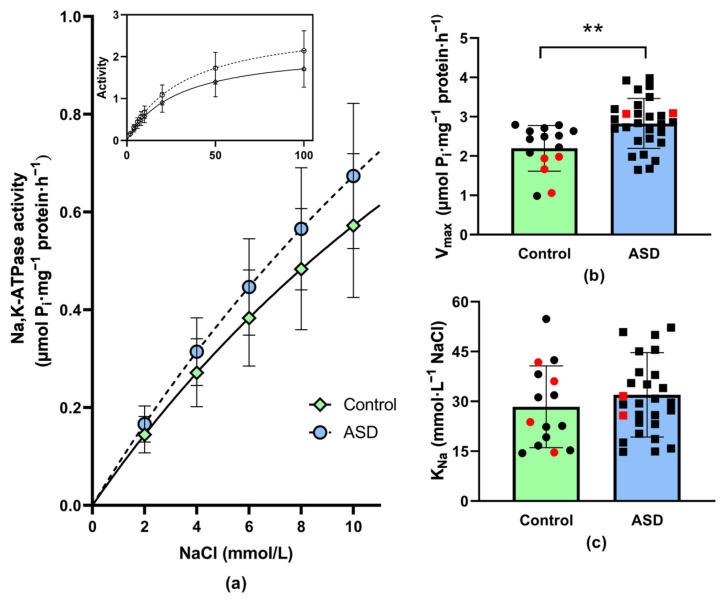
Na,K-ATPase enzyme activity as a function of NaCl concentrations ranging from 2 to 10 mmol/L. Inset—Na,K-ATPase enzyme activation in the whole investigated concentration range of NaCl (**a**). Kinetic parameters of Na,K-ATPase enzyme: maximal velocity of the enzyme reaction (V_max_) (**b**) and concentration of Na^+^ required to achieve the half-maximal velocity of the enzyme reaction (K_Na_) (**c**) in erythrocyte membranes of neurotypical (i.e., control) children (*n* = 15) and children with autism spectrum disorder (ASD, *n* = 28). ** *p* < 0.001 versus the control group. Data are presented as means ± standard deviations. Statistical significance between groups was assessed using the unpaired *t*-test. Red dots indicate female study participants.

When activating the Na,K-ATPase with an increasing concentration of sodium ions, the enzyme activity was higher in samples from ASD children as compared with control children. In the presence of the lowest applied concentration of NaCl (2 mmol/L), the activation of the enzyme was higher by 13% in the ASD group. At a higher presence of NaCl, the difference gradually increased, and in the presence of 100 mmol/L NaCl, the increase in enzyme activity amounted to 22% in the ASD group (Figure 3a).

The evaluation of the obtained data according to Michaelis–Menten equation resulted in a statistically significant increase in the V_max_ value by 25% in the ASD group (Figure 3b). The slight increase in K_Na_ (+11%) did not reach the level of statistical significance (Figure 3c).

## 4. Discussion

In this study, we aimed to assess the oxidative and antioxidative status in the blood plasma as well as in the RBCs of ASD individuals and neurotypical controls. Noteworthy, the same set of parameters reflecting possible oxidative stress was measured in both types of samples in order to effectively match and compare potential changes in the ASD condition.

### 4.1. Changes in Oxidative Stress Markers in Children with ASD

Protein oxidation, quantified by determining the AOPP concentration, has previously been shown to be higher in the plasma of ASD individuals, but also in siblings of children with ASD, compared with controls with no ASD relationship [32]. In the ASD children involved in our study, an AOPP increase was not significant in the plasma, while it was statistically significant in the RBCs, suggesting greater oxidation of RBC proteins than those proteins occurring in the blood plasma. A possible discrepancy in AOPP plasma concentration in children with ASD between the previously published study (where AOPP is reported in μmol per mL of plasma volume) [32] and our data may be due to the use of different units, as we adjusted the AOPP concentration to the plasma protein level.

The more intense RBC oxidative damage in ASD children proposed here was also demonstrated when focusing on lipid peroxidation. TBARS concentration was significantly higher in the RBCs but not in the plasma of our ASD children. These data support the idea that in ASD, the level of oxidative stress is higher in RBCs than at the systemic level, i.e., than that determined in blood plasma. No differences in AOPP and TBARS plasma concentrations between children with ASD and controls were described previously [33,34], while an increase in TBARS levels in RBCs in children with ASD was documented in [19], in line with our observations. Despite the statistically significant differences in the RBC concentrations of AOPP and TBARS, the assessment of antioxidant power by determining FRAP, as well as the general marker of oxidative stress, the GSH/GSSG ratio, showed that the antioxidant status is not completely disturbed in the RBCs of ASD children. The observed trend toward a lower GSG/GSSG ratio in the ASD group compared with the control group can be considered consistent with other studies that determined this ratio in the blood plasma of ASD individuals, where it was lower compared with controls [35,36,37,38].

Focusing on antioxidant enzymes, more data have already been published, but are often inconsistent. CAT activity was shown to be lower [34,39], higher [40], as well as unchanged [19] in the RBCs of ASD children in comparison with the controls. Our data showed that an increase in CAT activity did not reach the level of statistical significance in RBCs, but appeared highly significant in the blood plasma of ASD children when compared with neurotypical ones. This finding is in agreement with previously published enhanced circulating CAT activities in ASD patients [33], although a decrease in the activity of circulating SOD was reported in these patients as well, consistently with recently published data [41]. Although we did not notice modifications in SOD activity in ASD patients, either in the blood plasma or in the RBCs, our experiments support other observations also reporting no changes in SOD activity, either in the RBCs or in the plasma of ASD individuals [19,42,43]. In contrast, SOD activity in the RBCs of ASD patients has been found to be higher [34,39,40,44] as well as lower [45] compared with neurotypical individuals. In addition, another study reported age-related changes in RBC SOD activity. Before the age of 6 years, SOD activity was lower in ASD children, while it was not changed in older children [46]. This observation could at least partially explain the inconsistent findings published by different research teams, although we did not observe any correlation between the antioxidant enzyme activities and the age of participants. In the RBCs of ASD patients, we observed unchanged GPx activity, consistent with a previously published paper [47], although a decrease in this enzyme activity in RBCs has also been reported [34,45]. Conflicting results regarding GPx activity have been obtained by analyzing plasma samples as well—it has been found to be higher [42,44] and lower [45] in ASD individuals compared with controls, while our data support its increase in children with ASD. Since ASD represents a somewhat heterogenous group of neurodevelopmental disorders, it can be assumed that oxidative stress is not equally responsible for behavioral disturbances in different individuals.

### 4.2. Are Erythrocyte Properties Altered in Children with ASD?

To the best of our knowledge, there is no study reporting no differences between ASD and neurotypical individuals when focusing on RBCs. Oxidative stress was suggested to be at least partially responsible for the multiple morphological alterations to RBCs taken from ASD individuals, as incubation with an antioxidant significantly restored the RBC morphology [21]. Abnormal RBC morphology in ASD was also observed in another study [20]. Scanning electron microscopy was applied in both studies [20,21]. Our study revealed observable abnormalities in RBC membrane morphology using light microscopy, with evidently limited resolution compared to scanning electron microscopy. The formation of echinocytes, which was more frequent in our ASD children, may have been caused not only by intrinsic but also by extrinsic factors, e.g., due to contact with the glass surface [48], which should be taken into account. Nevertheless, since the handling of all blood samples was identical, we can hypothesize increased echinocyte formation due to intrinsic factors occurring in ASD. In line with these findings, the triad consisting of the observed alterations to RBC shape and cytoskeletal β-actin, together with oxidative modifications of the RBC membrane, was suggested to be a potential measurable marker for ASD [20]. The transition from a discoid shape to echinocytes was also observed during the storage of RBCs, simultaneously with an increase in their stiffness [49]. It was reported that the RBC rigidity increases progressively with the degree of echinocytosis [50]. This is consistent with the findings of our study showing an increase in osmotic resistance concomitant with a decrease in RBC deformability, indicating an increase in RBC membrane rigidity.

In the search for mechanisms that could be responsible for such differences in RBC mechanical properties between ASD and neurotypical children, the finding of increased oxidative damage to RBCs (discussed in the previous subsection) supports our observation of significantly higher levels of reactive oxygen species in RBCs from ASD children (visualized using the redox-sensitive fluorescent probe DCF). In addition, higher NO level in the RBCs of ASD children might also be responsible for RBC membrane alterations leading to a decrease in RBC deformability, as an adequate NO concentration was shown to be a prerequisite for optimal RBC deformability [51]. An enhancement in NO production by RBCs in ASD was also suggested previously, but only via the measurement of nitrate and nitrite concentrations [42].

Despite the observed statistically significant differences in RBC deformability, osmotic resistance, NO and reactive oxygen species, some RBC parameters were revealed to be equal in both the control and ASD groups. An increase in calcium ion concentration triggers the eryptosis, i.e., suicidal RBC death [52], which is not applicable in our participants with ASD. Similarly, the unchanged MCV and RDW parameters in ASD children do not support the theoretical idea that basic hematological analysis could help in the diagnosis of ASD.

### 4.3. Na,K-ATPase Enzyme Kinetics in Erythrocyte Membranes and ASD

Study of enzymes maintaining the proper intracellular ion concentration showed that changes in Na,K-ATPase activity are probably linked with ASD. Post-mortem studies of brain samples from children with autism (13 ± 3.7 years old) showed that this enzyme, with a crucial role in intercellular signal transmission via maintaining the optimal homeostasis of sodium and potassium ions, had higher activity in the frontal cortex and in the cerebellum compared with samples from age-matched (12.5 ± 3.5 years old) control subjects [53]. Another study of metabolic biomarkers in blood plasma indicated an impairment of energy metabolism in the brains of children with autism (3 to 15 years old), as indicated by a 40% increase in the lactate level compared with typically developing control children (unspecified age). In those individuals with ASD, the activity of Na,K-ATPase in RBCs was higher by more than 70% as compared to the control subjects [54], while these alterations observed in autistic children were accompanied by signs of oxidative stress [44]. In contrast, another study documented oxidative stress in the blood plasma of autistic children (aged 5 to 12 years) that showed significantly lower activity (by 66%) of the Na,K-ATPase enzyme in the RBCs of ASD compared with the RBCs of control children [19].

In all of the abovementioned studies focused on Na,K-ATPase, the enzyme activity was measured in the presence of certain specific conditions of ionic and substrate concentrations. In the present study, by varying the concentration of sodium within a wide range corresponding to physiologically relevant intracellular but also extracellular levels, we aimed to provide more detailed information concerning the alteration of Na,K-ATPase levels in the RBCs of ASD patients. Based on the results presented in our study, we can assume that the elevated enzyme activity in the RBCs of ASD children in all of the investigated concentrations of sodium was caused by the higher presence of active Na,K-ATPase molecules, as suggested by the increased V_max_ value. Our kinetic measurements also provide information concerning the ability of the enzyme to bind sodium. The similarities of the K_Na_ values in both groups of children exclude the possibility of larger structural changes in the sodium-binding region of the enzyme in the autistic as well as the control group.

### 4.4. Sex-Specific Differences

It is widely accepted that sex differences occur in the prevalence and manifestation of ASD, while reported male-to-female ratios range from 2:1 to 5:1 in favor of males [55,56,57]. In the available databases, studies are focused on the possible causes of this phenomenon [58,59,60,61], but so far without definitive conclusions. The low number of girls included in our study, as well as the unequal number of participating girls and boys, represent a significant limitation to present possible sex differences. However, it is worth mentioning that we did not observe clear sex-specific differences in the parameters that our study focused on. The only exception might be in the case of the number of active Na,K-ATPase molecules represented by the V_max_ value in the control group, with the tendency being for lower values in girls than in boys (Figure 3b).

Although we were unable to observe any signs of differences in oxidative stress markers between girls and boys, it was observed that the male brain is more susceptible to suspected neurotoxic substances and the female brain is better protected against oxidative damage [61]. Via the use of experimental animals, it was proposed that possible female protective factors could include a more efficient glutathione regeneration cycle and greater antioxidant capacity of the brain tissue [62]. However, this is an important area that should be explored further.

## 5. Conclusions

The obtained data indicate greater oxidative damage to proteins and lipids in the RBCs than to those in the blood plasma of ASD subjects. In addition, antioxidant enzyme activities (CAT and GPx) were increased in plasma samples of children with ASD compared with controls, while not in the RBCs. Changes in oxidative status could at least partially contribute to the deterioration of RBC deformability observed in children with ASD. Moreover, significantly higher NO levels and Na,K-ATPase enzyme activity were detected in the RBCs of ASD individuals in comparison with neurotypical children. Abnormalities in the antioxidant status and RBC properties may be involved in the pathomechanisms of ASD and contribute to its clinical manifestations.

## Figures and Tables

**Table 1:** Parameters of oxidative stress and antioxidant status determined in blood plasma and RBCs of neurotypical (i.e., control) children and children with ASD.

PLASMA	Control (*n* = 14–17)	ASD (*n* = 18–28)	Statistics (*p* Value)
AOPP (μmol/g prot)	0.664 ± 0.3	0.998 ± 0.73	0.11
FRUC (mmol/g prot)	0.017 ± 0.003	0.022 ± 0.003	**0.015**
TBARS (μmol/L)	1.55 ± 0.3	1.62 ± 0.34	0.56
FRAP (μmol/L)	570 ± 81	536 ± 100	0.24
GSH/GSSG	3.34 ± 0.29	3.01 ± 0.45	0.06
CAT (U/mL)	0.636 ± 0.35	1.21 ± 0.39	**<0.0001**
SOD (inhibition rate %)	2415 ± 232	2392 ± 234	0.77
GPx (U/L)	139 ± 21	155 ± 25	**0.046**
**ERYTHROCYTES**	**Control** **(*n* = 15–17)**	**ASD** **(*n* = 29–31)**	**Statistics (*p* Value)**
AOPP (μmol/g Hb)	7.25 ± 0.9	7.88 ± 0.8	**0.015**
FRUC (mmol/g Hb)	3.49 ± 0.67	3.73 ± 0.94	0.37
TBARS (μmol/L)	63.4 ± 22.3	93.7 ± 38.6	**0.007**
FRAP (μmol/L)	44,937 ± 4636	45,771 ± 5595	0.61
GSH/GSSG	0.68 ± 0.07	0.71 ± 0.15	0.43
CAT (U/mg Hb)	5.09 ± 0.67	5.52 ± 0.77	0.06
SOD (inhibition rate %)	830 (590; 2660)	710 (500; 880)	0.15
GPx (U/g Hb)	155 ± 46	160 ± 47	0.73

Abbreviations: ASD—autism spectrum disorder, AOPP—advanced oxidation protein products, FRUC—fructosamine, TBARS—thiobarbituric acid reactive substances, FRAP—ferric reducing antioxidant power, GSH/GSSG—the ratio of reduced to oxidized glutathione, CAT—catalase, SOD—superoxide dismutase, GPx—glutathione peroxidase, Hb—hemoglobin. Data are presented as means ± standard deviations or as median with interquartile range where appropriate. Statistical significance between groups was assessed using the unpaired *t*-test when data were normally distributed or the Mann–Whitney test when the distribution was non-Gaussian. The *p*-values in bold are statistically significant.

**Table 2:** Erythrocyte morphology in children with ASD and neurotypical controls.

Erythrocyte Shape	Control (*n* = 10)	ASD (*n* = 7)
Normal	95.1%	81.6%
Echinocyte I	4.4%	15.7%
Echinocyte II	0.5%	2.7%

ASD—autism spectrum disorder. Data are presented as the percentage of normal erythrocytes and echinocytes (echinocyte I—irregularly contoured erythrocytes; echinocyte II—erythrocytes with spicules) from the total erythrocyte count, while an average of 750 erythrocytes were analyzed from each individual.

## Data Availability

The data supporting the findings of this study are available in this article.
